# A Novel Recombinant Peste des Petits Ruminants-Canine Adenovirus Vaccine Elicits Long-Lasting Neutralizing Antibody Response against PPR in Goats

**DOI:** 10.1371/journal.pone.0037170

**Published:** 2012-05-18

**Authors:** Junling Qin, Hainan Huang, Yang Ruan, Xiaoqiang Hou, Songtao Yang, Chengyu Wang, Geng Huang, Tiecheng Wang, Na Feng, Yuwei Gao, Xianzhu Xia

**Affiliations:** 1 College of Animal Science and Veterinary Medicine, Agricultural Division, Jilin University, Changchun, People’s Republic of China; 2 Veterinary Institute, Academy of Military Medical Sciences, Changchun, People’s Republic of China; 3 College of Animal Science and Technology, Jilin Agricultural University, Changchun, People’s Republic of China; 4 Academy of Animal Science and Veterinary Medicine of Jilin Province, Jilin, Changchun, People’s Republic of China; Mayo Clinic, United States of America

## Abstract

**Background:**

Peste des petits ruminants (PPR) is a highly contagious infectious disease of goats, sheep and small wild ruminant species with high morbidity and mortality rates. The Peste des petits ruminants virus (PPRV) expresses a hemagglutinin (H) glycoprotein on its outer envelope that is crucial for viral attachment to host cells and represents a key antigen for inducing the host immune response.

**Methodology/Principal Findings:**

To determine whether H can be exploited to generate an effective PPRV vaccine, a replication-competent recombinant canine adenovirus type-2 (CAV-2) expressing the H gene of PPRV (China/Tibet strain) was constructed by the *in vitro* ligation method. The H expression cassette, including the human cytomegalovirus (hCMV) promoter/enhancer and the BGH early mRNA polyadenylation signal, was inserted into the *SspI* site of the E3 region, which is not essential for proliferation of CAV-2. Infectious recombinant rCAV-2-PPRV-H virus was generated in transfected MDCK cells and used to immunize goats. All vaccinated animals produced antibodies upon primary injection that were effective in neutralizing PPRV *in vitro*. Higher antibody titer was obtained following booster inoculation, and the antibody was detectable in goats for at least seven months. No serious recombinant virus-related adverse effect was observed in immunized animals and no adenovirus could be isolated from the urine or feces of vaccinated animals. Results showed that the recombinant virus was safe and could stimulate a long-lasting immune response in goats.

**Conclusions/Significance:**

This strategy not only provides an effective PPR vaccine candidate for goats but may be a valuable mean by which to differentiate infected from vaccinated animals (the so-called DIVA approach).

## Introduction

Peste des petits ruminants (PPR) is an acute and highly contagious disease of small ruminants, particularly affecting goats and sheep. Disease onset is characterized by fever and somnolence; within a few days, mucosal erosions occur, accompanied by oral and ocular discharge, diarrhea, and symptoms of pneumonia. The disease may resolve after a prolonged convalescent period, but more frequently leads to death [1]. PPR was first described in 1942 in Côte d’Ivoire as a fatal disease of small ruminants that resembled rinderpest of cattle. Since then, PPR has spread throughout west and sub-Saharan Africa, the Middle East, the Indian subcontinent, Turkey, some countries in Central Asia, and China [2]–[9]. Currently, PPR is responsible for significant economic losses in goats and sheep productivity in the endemic regions.

PPR is caused by Peste des petits ruminant virus (PPRV), a member of the genus Morbillivirus, family Paramyxoviridae [10], [11]. PPRV, like other morbilliviruses, is an enveloped, nonsegmented negative-strand RNA virus. PPRV expresses two external glycoproteins, fusion (F) and hemagglutinin (H), which mediate infectivity. The H protein binds to its cognate receptor on the host cell during the first step of the viral infection process; as such, it acts as a major antigen that stimulates a protective immune response in the host [12]–[14].

PPR has been controlled for many years by the use of a rinderpest virus (RPV) tissue culture-adapted vaccine [15]. However, the remarkable success of the global rinderpest eradication program has led to the RPV vaccine being banned from use within rinderpest-free zones; the fear is used in any species, including the PPRV-susceptible small ruminants, would hinder the establishment of the serologically negative population.

In response to this ban, a homologous PPR vaccine was produced by passaging the Nigeria 75/1 strain of PPRV 63 times in Vero cells [16], and is now being introduced into some PPR-endemic regions. While this vaccine produces a sufficient PPRV neutralizing response, it is not optimal for two main reasons. First, PPRV, like all members of the family Paramyxoviridae, is very heat sensitive and its endemic areas are nearly all in hot climates. Second, the live attenuated PPRV vaccine generates a broad range of immune responses to viral proteins, making it difficult to serologically distinguish vaccinated animals from those naturally infected. Ultimately, sero-epidemiosurveilance of the disease is impossible in endemic areas where a vaccination program has been or is being implemented [17]. Thus, the live attenuated tissue-culture approach needs to be replaced by a vaccine strategy that will facilitate differentiating infected from vaccinated animals, the so-called “DIVA” vaccines [18].

Over the past decade, adenoviruses have emerged as particularly promising vaccine delivery vehicles. They are double-stranded DNA viruses with high genetic stability, exhibiting no mutations or insertion/deletions after multiple rounds of replication *in vitro*. Furthermore, they do not integrate into the genome of the infected host, and consequently present less of a safety concern for gene delivery since there is little risk of insertional mutagenesis and expression of potentially toxic or deleterious gene products [19]–[21]. Due to their nonenveloped structure, adenoviruses are physically stable and can withstand lyophilization, suggesting a convenient means for storage, transport, and vaccine formulation that is amenable to global distribution.

Two serotypes of canine adenovirus (CAV) have been isolated. Both CAV-1 and CAV-2 are associated with mild upper respiratory tract infections in dogs, and effective and safe live-modified vaccines are available for routine vaccination of dogs against both. The structure, replication, and transcription of CAV-2 are very well-characterized and it has been more extensively utilized for vector development [22]. When foreign genes are inserted into the non-essential E3 region of CAV-2, replication of the recombinant virus is not impacted, resulting in high virus titers and high-level gene expression [23], [24]. Here, we describe the development of a recombinant canine adenovirus type-2 delivery vector expressing the H gene of PPRV and its utility as a vaccine in experimental goats.

## Results

### Identification of Recombinant virus CAV-2-H

**Figure 1:**
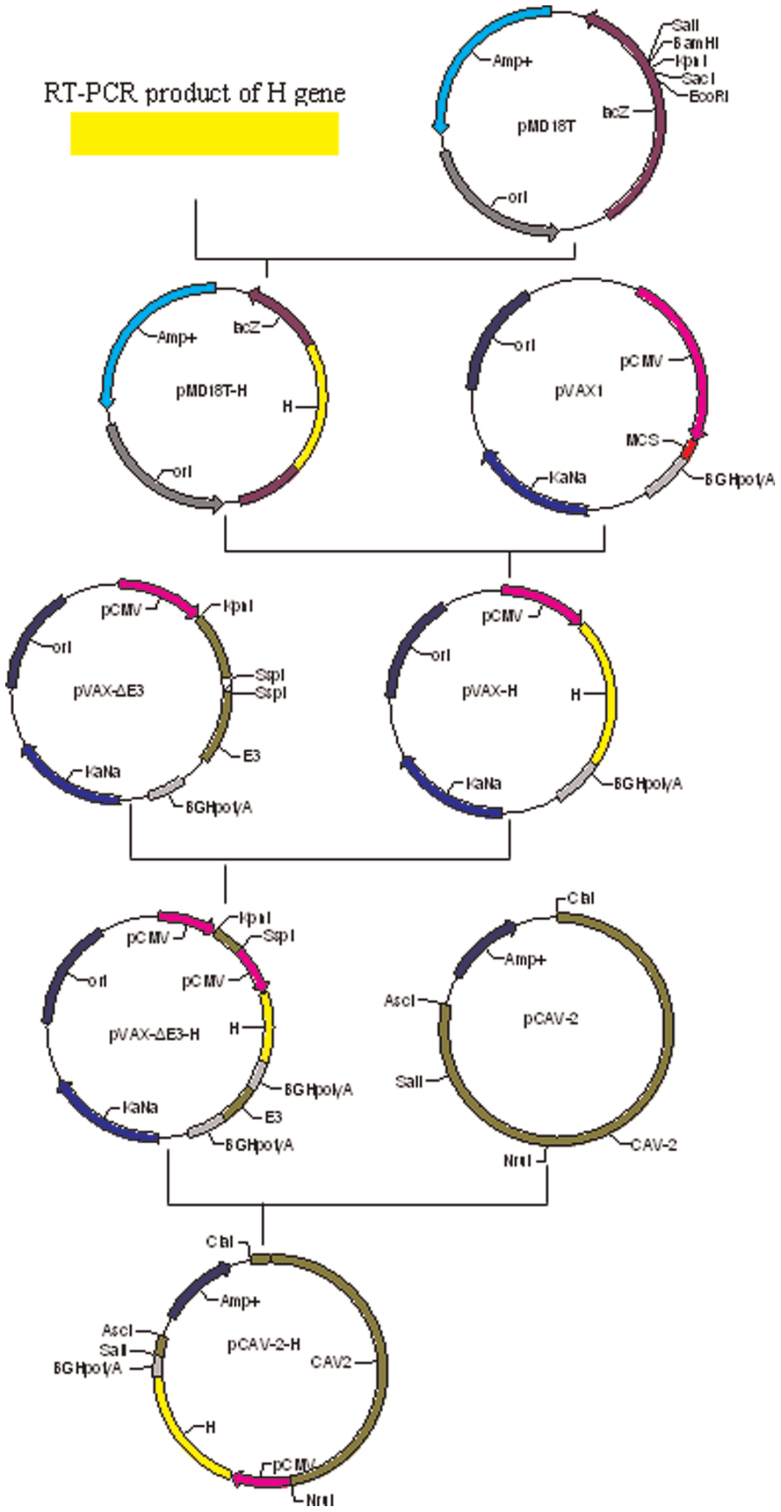
Schematic diagram showing the construction strategy of the recombinant virus rCAV-2-PPRV-H. Cassette expression is under the control of the CMV promoter.

We successfully generated a recombinant virus rCAV-2-PPRV-H expressing the PPRV H protein. The H gene expression cassette was subcloned into the E3 region of the pPolyII-CAV-2 plasmid, generating a recombinant genome pPolyII-CAV-ΔE3-H (Fig. 1). The identification of the plasmid pVAX-H, pVAXΔE3-H and pPolyII-CAV-ΔE3-H by restriction endonuclease digestion confirmed that the H gene and its expression cassette were included in the recombinant virus (data not shown). Eleven days after transfection, typical CPE (grape-cluster-like cells) was observed under a microscope. Adenovirus-like particles were observed under the electron microscope. The growth characteristics of the recombinant virus were similar to that of the canine adenovirus vaccine strain YCA18. The TCID_50_ of the rCAV-2-PPRV-H virus was assayed and calculated to be 10^7.8^/mL. This virus hemagglutinates human red blood cells and has an HA titer of 1∶2^9^, which is similar to the titer of the canine adenovirus vaccine strain YCA18. The genome of rCAV-2-PPRV-H was analyzed for genetic stability by restriction enzyme analysis and found to retain the correct genome structure at the 30th serial passage (data not shown).

### Western Blotting

**Figure 2:**
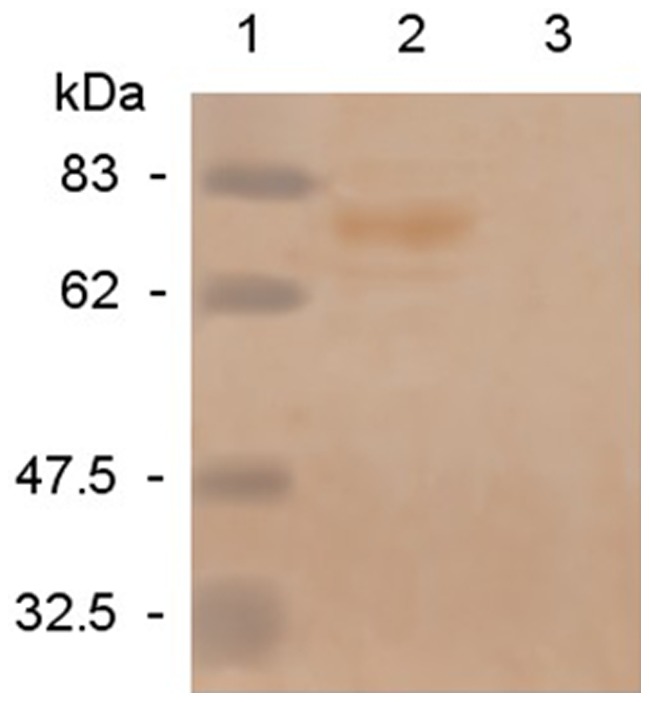
Western blotting analysis of the PPRV H produced in MDCK cells infected with rCAV-2-PPRV-H or CAV-2. Lanes: 1, MV Marker; 2, MDCK cells infected with rCAV-2-PPRV-H; 3, MDCK cells infected with CAV-2.

Recombinant adenovirus-infected MDCK cell lysates were examined by Western blotting and compared with results from the negative control canine adenovirus vaccine strain YCA18. Immunoreactivity of H protein (approximately 70 kD) was found in recombinant adenovirus-infected cell lysates, but not in vaccine strain YCA18 (Fig. 2), indicating that the expressed H protein retained its antigenic reactivity.

At passage 30, the results of RT-PCR detection of H protein of PPRV and Western blotting analysis of H protein expressed by recombinant virus were the same (data not shown). All results indicated that the recombinant virus was stable enough to maintain its genetic integrity.

### Immunofluorescence

**Figure 3:**
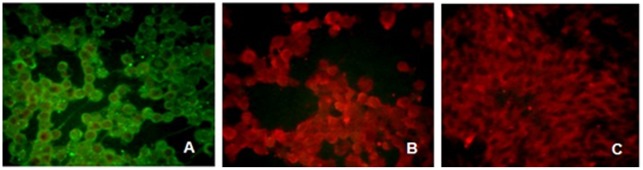
Fluorescence microscopy analysis of PPRV H protein in recombinant virus-infected MDCK cells. MDCK cells were infected with rCAV-2-PPRV-H (A) or CAV-2 (B) at 1 m.o.i. Negative controls were untransfected cells (C). After 48 h, the infected cells were detected with goat anti-PPRV antiserum, followed by Alexa Fluor® 488-conjugated donkey anti-goat IgG and observed under fluorescence microscope.

The cellular localization of the H protein expressed by the recombinant adenovirus was investigated immunologically using fixed MDCK cells. H protein expression was readily detected on the surface of MDCK cells infected with CAV-2-PPRV-H (Fig. 3).

### Antibody Assays

In test goats (The designs of the animal experiments are shown in Table 1), neutralizing antibody titers against PPRV at week 3 after the primary vaccination ranged from 1∶32–1∶64 with rCAV-2-PPRV-H, 1∶64–1∶128 with the attenuated PPR vaccine, and <1∶2 with CAV-2 injection. At weeks 5–6 (two to three weeks after the booster injection) goats who received rCAV-2-PPRV-H exhibited the highest neutralizing titers against H protein. At thirty weeks after primary injection, at the end of the trial, titers were still above the protective level of 1∶10, which indicated that the antibody may persist for a longer time. Comparatively, neutralizing antibody titers induced by the attenuated PPR vaccine reached higher levels than those induced by rCAV-2-PPRV-H from week 2 after the primary vaccination and onward (Table 2).

**Table 1:** Experimental designs of the animal studies.

Group	Vaccine	Route	Dosage	Dose
A-1(n = 35)	rCAV-2-PPRV-H	IM	10^7.8^ TCID_50_	2 doses, 3 weeks interval
A-2(n = 21)	rCAV-2-PPRV-H	IM	10^7.8^ TCID_50_	2 doses, 3 weeks interval
B(n = 3)	Attenuated PPRV	IM	10^7.8^ TCID_50_	2 doses, 3 weeks interval
C(n = 3)	CAV-2	IM	10^8^ TCID_50_	2 doses, 3 weeks interval

IM stands for intramuscular.

**Table 2:** Neutralizing Antibody titers against PPRV in goats immunized with attenuated PPRV vaccine, rCAV-2-H and CAV-2.

Group	Weeks post-primary vaccination										
	0	3	6	9	12	15	18	21	24	27	30
A-1^a^	<1∶2	1∶32–1∶64	1∶64–1∶128	1∶64–1∶128	-	-	-	-	-	-	-
A-2^b^	<1∶2	1∶32–1∶64	1∶64–1∶128	1∶64–1∶128	1∶64–1∶128	1∶64–1∶128	1∶64–1∶128	1∶32–1∶64	1∶32–1∶64	1∶32–1∶64	1∶32–1∶64
B^c^	<1∶2	1∶64–1∶128	1∶128–1∶256	1∶128–1∶256	1∶128–1∶256	1∶128–1∶256	1∶64–1∶128	1∶64–1∶128	1∶64–1∶128	1∶64	1∶64
C^d^	<1∶2	<1∶2	<1∶2	<1∶2	<1∶2	<1∶2	<1∶2	<1∶2	<1∶2	<1∶2	<1∶2

The level of serum PPRV-specific IgG in goats was determined by VNA, and the serum samples were collected at weeks 0–30 following the primary vaccination. ^ab^ *P*>0.05, ^ac^ *P*<0.05, ^bc^ *P*<0.05.

The HI antibodies against CAV-2 continually increased from week 2 after vaccination, which corresponded with the appearance of PPRV neutralizing antibody; however, the increase of the PPRV neutralizing antibody was more gradual. The highest HI titer of the goat serum was 1∶1024, which dropped to 1∶64 by week 30 (Table 3).

**Table 3:** HI antibody titers of CAV-2 in goats immunized with attenuated PPRV vaccine, rCAV-2-H and CAV-2.

Group	Weeks post-primary vaccination										
	0	3	6	9	12	15	18	21	24	27	30
A-1	<1∶2	1∶2^5^–1∶2^6^	1∶2^8^–1∶2^10^	1∶2^9^–1∶2^10^	–	–	–	–	–	–	–
A-2^a^	<1∶2	1∶2^5^–1∶2^6^	1∶2^8^–1∶2^10^	1∶2^9^–1∶2^10^	1∶2^8^–1∶2^10^	1∶2^8^–1∶2^10^	1∶2^7^–1∶2^9^	1∶2^7^–1∶2^8^	1∶2^6^–1∶2^7^	1∶2^6^–1∶2^7^	1∶2^5^–1∶2^6^
B^b^	<1∶2	<1∶2	<1∶2	<1∶2	<1∶2	<1∶2	<1∶2	<1∶2	<1∶2	<1∶2	<1∶2
C^c^	<1∶2	1∶2^5^–1∶2^6^	1∶2^9^–1∶2^10^	1∶2^9^–1∶2^10^	1∶2^9^–1∶2^10^	1∶2^9^–1∶2^10^	1∶2^7^–1∶2^9^	1∶2^7^–1∶2^9^	1∶2^6^–1∶2^7^	1∶2^6^–1∶2^7^	1∶2^6^

ab *P*<0.05, ^ac^ *P*>0.05, ^bc^ *P*<0.05.

### Lymphocyte Proliferation Responses

All the test groups of goats produced robust lymphoproliferative responses, as compared to the PBS control (*P*<0.05); however, there was no significant difference in the responses between the goats immunized with attenuated PPRV vaccine, rCAV-2-PPRV-H, or CAV-2 (*P*>0.05) (Table 4).

**Table 4:** Proliferative responses of PBMC of goats immunized with attenuated PPRV vaccine, rCAV-2-PPRV-H, CAV-2 and PBS.

Group	Mean stimulation index±SD
Attenuated PPRV vaccine^a^	2.89±0.27
rCAV-2-PPRV-H^b^	2.75±0.38
CAV-2^c^	2.65±0.41
PBS^d^	1.18±0.26

Lymphocyte proliferative responses were analyzed by using PBMC collected at the day three weeks after boosting. ^ab^ *P*>0.05, ^ac^ *P*>0.05, ^bc^ *P*>0.05, ^ad^ *P*<0.05, ^bd^ *P*<0.05, ^cd^ *P*<0.05.

## Discussion

Attenuated canine adenovirus type 2 has proved to be an effective vaccine strain used for prevention of canine hepatitis and infectious laryngotrachitis. Although canine adenovirus is a natural pathogen of dogs, it replicates efficiently in goats following inoculation by various routes, including subcutaneous and intramuscular (in our preliminary experiment). Attenuated CAV-2 has also been shown to be safe in other animals, including cats, foxes and swine [25]–[27]. As with human adenoviruses, a CAV-2 expression vector with a deleted E3 has some advantageous features for gene delivery applications: it replicates efficiently to high titers, provides large cloning space, permits the expression of recombinant proteins in most mammalian cell lines and tissues, and accurately expresses and modifies recombinant proteins. Another important advantage of CAV-2 is the fact that as a natural canine pathogen there are no pre-existing, maternally-derived antibodies in livestock, which could otherwise interfere with vaccine efficacy in young and growing goats.

As a feasibility study, evaluation of the safety of the vaccine is an important concern. Regardless of efficacy, a recombinant virus must cause minimal to no adverse effects in experimental animals. The rCAV-2-PPRV-H could not be isolated from the urine or feces of vaccinated goats, up to the end of the monitoring period on day 35 post-inoculation. This finding further indicated that the recombinant virus could not be shed through urine and feces of immunized goats to contaminate the environment during this period. In another experiment (data not shown), we checked the horizontal infection rates of the recombinant virus by evaluating goats in close contact (living in the same yards) with vaccinated goats; antibodies against CAV-2 or PPRV were not detected in the in-contact control animals, further ruling out the possibility of rCAV-2-PPRV-H shedding from experimental animals with the recombinant virus.

In order to analyze which immunization route could be better and more efficient to enhance high level neutralizing antibody, we also investigated different routes of administration for the recombinant virus. However, the only effective route of inoculation for eliciting neutralizing antibodies against PPRV in goats was intramuscular injection. The oral inoculation and intranasal administration did not produce any antibodies against either adenovirus or PPRV (data not shown), indicating that no effective immune reaction can be elicited by these two routes. This suggested that goats could not be naturally infected with CAV-2, especially since there was no antibody against CAV-2 in goats before administration. Avoiding interference of pre-existing antibodies is an important requirement for successful recombinant vaccine; the fact that there is no chance of pre-existing antibody interference to CAV-2 in goats enhances the potential of this adenovirus vaccine to stimulate a strong and efficient immune response.

The hemagglutinin protein of PPRV has been shown to play a crucial role in stimulating antibody responses against PPR infection [28]. According to standards of OIE, a VNT of 10 is the minimum value for quality control and efficacy evaluation for the PPR-like attenuated vaccine, Nig/75/1 [29]. In order to enhance the level of immune responses of rCAV-2-PPRV-H in our experimental goats, immunizations with rCAV-2-PPRV-H were carried out twice with the same dose at 21-day intervals. The titers of neutralizing antibody against PPRV induced by rCAV-2-PPRV-H were significantly enhanced after booster of rCAV-2-PPRV-H. A single immunization of rCAV-2-PPRV-H induced the antibody against PPRV up to 1∶32-1∶64 within three weeks. After the booster, a titer of 1∶64–1∶128 was achieved, which surpassed the minimum required level for PPRV by a considerable margin.

Although the antibody response against PPRV has largely been considered the most important factor in efficient protection, good cellular immunity stimulated by a vaccine would also enhance this efficiency and persistence, as well as induce more durable immunity. Therefore, we also investigated the cell-mediated immune response in goats by lymphocyte proliferation assay. Live attenuated vaccines are known to be efficient in inducing long-lasting immunity *via* cellular and humoral immune responses [30], [31]. The data from the lymphocyte proliferation assay showed that, as compared with the negative control, the three groups immunized with attenuated PPRV, rCAV-2-PPRV-H, and CAV-2 stimulated similar high cellular immune responses; in addition, the cell-mediated immunity efficacies between the attenuated PPRV group and rCAV-2-PPRV-H group were nearly identical. Even if the VNA titer of the recombinant virus vaccinated animal is undetectable, the animal may still be protected against PPRV challenge [32]. This further demonstrated that cell-mediated immunity plays a very important role in PPR vaccines.

In summary, a novel recombinant canine adenovirus expressing the H protein of PPRV was constructed, and the recombinant virus rCAV-2-PPRV-H expressed the H protein efficiently and induced humoral and cellular immunity against PPRV in goats. This adenovirus vector might be an attractive candidate DIVA vaccine for preventing the disease associated with PPRV infection and facilitating sero-epidemiosurveilance of PPR.

## Materials and Methods

### Cells, viruses, Genes and Plasmids

Madin-Darby canine kidney (MDCK) cells (American Type Culture Collection (ATCC), CCL-34) were cultured in Dulbecco’s Modified Essential Medium (DMEM; Invitrogen, USA) containing 10% (v/v) heat-inactivated fetal bovine serum (FBS; Gibco, USA), penicillin (100 U/mL) and streptomycin (100 mg/mL). Eighty percent confluent cells were used for transfection with the recombinant genome and fully confluent cells were infected with the recombinant virus for propagation and production. Vero cells (ATTC, CCL-81) were cultured in Minimum Essential Medium (MEM; Gibco) containing 10% FBS. Live attenuated PPRV vaccine strain Nigeria/75/1 (Nig/75/1) was obtained from the China Institute of Veterinary Drug Control. CAV-2 strain YCA 18 was isolated by Xia *et al*. [33]. A complete PPRV cDNA derived from a PCR product of PPRV strain China/Tibet/Geg/07-30 (GenBank accession no. FJ905304) was kindly provided by the China Animal Health and Epidemiology Center. Plasmid pPOLYII-CAV-2, containing the whole genome of canine adenovirus type-2, was constructed and described by Zhang *et al*. [34]; it was chosen for use here based on its features as an infectious plasmid that can produce canine adenovirus particles after transfection of MDCK cells.

### The Construction of pPolyII-CAV-ΔE3-H

The open reading frame (ORF) of the PPRV glycoprotein gene H was amplified by RT-PCR using the following primer pair, which introduced *KpnI* and *XhoI* restriction enzyme sites at the respective 5′-termini (underlined): forward primer H1, 5′-**CCG** GTA**CC**ATG**G**CCGCACAAAGGGAAAG-3′ (the complete Kozak sequence in bold); reverse primer H2, 5′-GCGCTCGAGTCAGACTGGATTACATGTTACCTC-3′. The PCR product of the H gene was subcloned into the pMD18-T vector (TaKaRa, China), and the resultantpMD18-T-H vector was sequenced by the UN Corporation of Shanghai, China. The 4.8 kb *KpnI* fragment containing the E3 region from pPolyII-CAV-2 was first cloned into pVAX1 (Invitrogen) to generate pVAX-ΔE3. The H sequence was released with *KpnI* and *XhoI* from pMD18-T-H and cloned into the *KpnI/XhoI* sites of pVAX1 to generate pVAX-H. The *MluI*/*HaeII* fragment of pVAX-H, containing the H cDNA expression cassette driven by the cytomegalovirus (CMV) immediate early promoter with BGH polyadenylation signals at the other end of the gene, was blunted using a DNA blunting kit (TaKaRa) and directionally cloned into the *SspI* site of pVAX-ΔE3, forming the pVAXΔE3-H vector. The *NruI* and *SalI* double digest pVAXΔE3-H fragment containing the H expression cassette flanked with residual E3 sequences was cloned back into pPolyII-CAV-2 by replacing the fragment between the *NruI* and *SalI* sites, forming the final pPolyII-CAV-ΔE3-H plasmid (Fig. 1). The recombinant genome was purified for use in transfection. The preparation, cloning and identification of genes and recombinant plasmids were performed according to standard methods [35].

### Transfection of the Recombinant Genome in MDCK Cells and Production of Recombinant virus

MDCK cells were transfected with recombinant genome pPolyII-CAV-ΔE3-H using Lipofectamine2000™ (Invitrogen), according to the manufacturer’s protocol. Briefly, MDCK cells (2.5×10^5^ cells/well) were cultured in six-well tissue culture plates (Nunc, USA). At approximately 70–80% confluence, supernatant medium was removed and cells were washed with OPTI-MEM (Gibco). Four micrograms of purified linearized recombinant virus genome were dissolved in 250 µL OPTI-MEM, and then mixed with 10 µL Lipofectamine2000™ dispersed in 250 µL OPTI-MEM and incubated at room temperature for 20 min. The transfection mix was then spread over the 80% confluent MDCK cells and incubated for six hours, when it was replaced by 2 mL complete DMEM (5% FBS). Transfected cells were incubated at 37°C and blind-passaged until cytopathic effects (CPE) appeared, when samples of cell lysates were collected for electron microscopic examination using negative staining with potassium phosphotungstate.

### Western Blotting

The supernatant of the recombinant virus cell culture lysates was separated on 15% SDS-PAGE gels and the proteins were transferred onto a nitrocellulose membrane (Bio-Rad, USA). The proteins were probed with goat anti-PPRV antiserum (1∶200) and horseradish peroxidase-labeled donkey anti-goat IgG antibody (1∶5000; Sigma, USA), according to the protocols described elsewhere [36].

### Immunofluorescence Analysis

To analyze expression of H protein on the surface of cells, MDCK cells were infected with CAV-2, rCAV-2-H, at a multiple of infection (m.o.i.) of 1 for 48 h. Afterwards, infected cells were fixed with 4% paraformaldehyde for 20 min at room temperature and treated with 0.1% Triton-X100. After washing three times with PBS, cells were incubated with goat anti-PPRV antiserum (1∶200), followed by Alexa Fluor® 488-conjugated donkey anti-goat IgG (1∶1000; Invitrogen). Surface expression of PPRV H was determined using a fluorescence microscope (BX51FL; Olympus, Japan).

### Animal Inoculation

A total of sixty-two outbred goats (6–24 months of age) were used for animal study. All goats had no detectable levels (titers <1∶2) of PPRV neutralizing antibodies or CAV hemagglutination-inhibition antibodies. To investigate the immunogenicity of rCAV-2-PPRV-H in goats, thirty-five goats were randomly assigned to group A-1 and received an intramuscular injection of 10^7.8^ TCID_50_ rCAV-2-PPRV-H. Twenty-one goats in group A-2 were used to investigate the PPRV VNA duration in goats immunized with rCAV-2-PPRV-H. Three goats were randomly assigned to group B and were intramuscularly inoculated with 10^7.8^ TCID_50_ attenuated PPRV vaccine. The remaining three goats made up group C (the negative control group) and received intramuscular injection of 10^8^ TCID_50_ CAV-2. All three groups received booster injection of the original dosage at three weeks after primary immunization. Every 3 weeks after primary immunization, animals were bled from the jugular vein. Sera were separated and stored at -20°C until analyzed.

The animal experiments were conducted with prior approval from the Animal Welfare and Ethics Committee of the Veterinary Institute at the Academy of Military Medical Sciences, China under the permit number (SCXK-2002-018). All manipulation of the goats satisfied the requirements of the Regulations of Experimental Animal Administration of PR China.

For one month after vaccination, all goats were monitored daily for clinical signs. Urine and feces samples were collected from goats inoculated with rCAV-2-PPRV-H and CAV-2 every two days for the first month. The samples were suspended in DMEM (5% FBS, 200 U/mL penicillin and 200 mg/mL streptomycin) and centrifuged at low speed to remove particulates. Supernatants were collected and used to inoculate MDCK cell monolayers, according to the previously described protocol [37].

### Canine Adenovirus Hemagglutination Inhibition (HI)

Canine adenovirus type-2 HI antibody titer was assayed according to the published protocol [38]. Briefly, goats from different groups were vaccinated with attenuated PPRV vaccine, rCAV-2-PPRV-H, or CAV-2 and the serum samples were collected at weeks 0-30 following the primary vaccination. Serial 2-fold dilutions of sera were mixed with 25 µL CAV-2, titer 4 HA units, with PBS (0.01 M, pH 7.2) as the control, and incubated at 37°C for 30 min. Then, 25 µL 1% human group O red blood cells suspended in PBS (pH 7.2) were added to each well and incubated at 37°C for 1 h. Titers were determined as the highest dilution showing at least partial agglutination.

### Virus Neutralization Test (VNT)

VNT was performed according to the recommendations from the World Organization for Animal Health (formerly, the Office International des Epizooties (OIE)). Briefly, the vaccine strain of PPRV (Nig 75/1) was grown in Vero cells. Aliquots of virus (100 TCID_50_) were incubated at 37°C for one hour with serial dilutions of the test or control sera (heat inactivated at 56°C for 30 min) and then were added to Vero cells growing in a flat-bottom 96-well tissue culture plate. Cells were observed microscopically for CPE starting at day 3 post-infection. The CPE recorded at day 5 was used for the calculation of PPRV virus neutralization (VNA) titers. Virus neutralization titer was defined as the highest serum dilution that inhibited CPE by at least 50%.

### Lymphocyte Proliferation Assay

Individual goats from experimental groups were bled from the jugular vein at week 3 after the booster injection. *Peripheral blood mononuclear cells* (PBMCs) were prepared as previously described [39]. Cells (10^6^ cells/well) were plated in 96-well plates, in triplicate, and were challenged with 5 µg/mL of concanavalin A for 48 h at 37°C in 5% CO_2_. The proliferative response was determined by MTS assays using the Cell Titer 96® aqueous one solution cell proliferation kit (Promega) and following the manufacturer’s instructions. Uninfected cells cultured in medium alone were used as negative controls. The lymphocyte proliferation rate was quantified by the stimulation index (SI), which was calculated as the ratio of OD_490 nm_ of stimulated cells to OD_490 nm_ of negative controls.

### Statistical Analysis

All data were conducted by two-way analysis of variance (ANOVA) and Student’s *t-*test, using SPSS v.13.0 software. A *P*-value of less than 0.05 was considered statistically significant.
